# Supplementary material for: A sequential EMT-MET mechanism drives the differentiation of human embryonic stem cells towards hepatocytes
Source: Nat Commun. 2017 May 3;8:15166. doi: 10.1038/ncomms15166 (PMC5418622; doi:10.1038/ncomms15166)
Supplement: Supplementary Information — Supplementary Figures and Supplementary Tables [file ncomms15166-s1.pdf]

Supplementary Figure 1

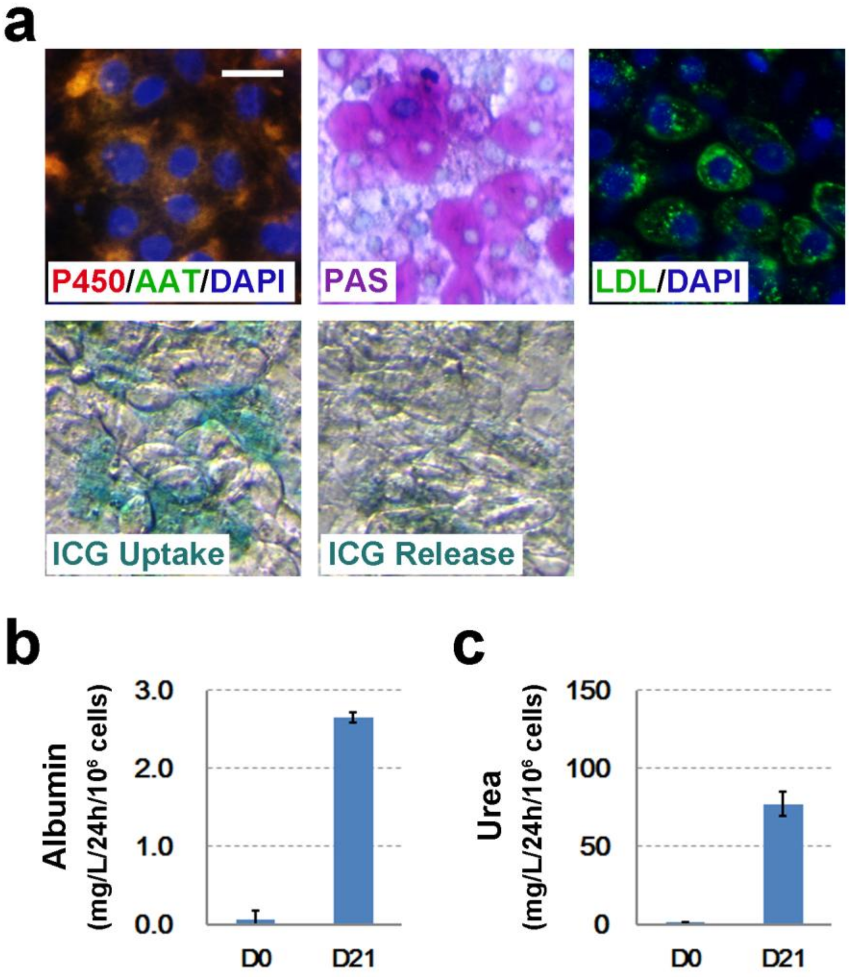

Supplementary Figure 1. Functional assays of hepatocyte-like cells induced from H1 hESCs

(a) P450 and AAT staining, PAS staining, LDL uptake assay, and ICG uptake and release assay for hepatocyte-like cells. Scale bar is 20 μm.

(b) Quantification of ALB secretion for undifferentiated H1 hESCs and H1 hESCs-derived hepatocyte-like cells. Data represent mean ± s.d. from three independent repeats (P=0.000048 when determined by Student's unpaired t-test).

(c) Quantification of urea secretion for undifferentiated H1 hESCs and H1 hESCs-derived hepatocyte-like cells. Data represent mean ± s.d. from three independent repeats (P=0.0036 when determined by Student's unpaired t-test).

Supplementary Figure 2

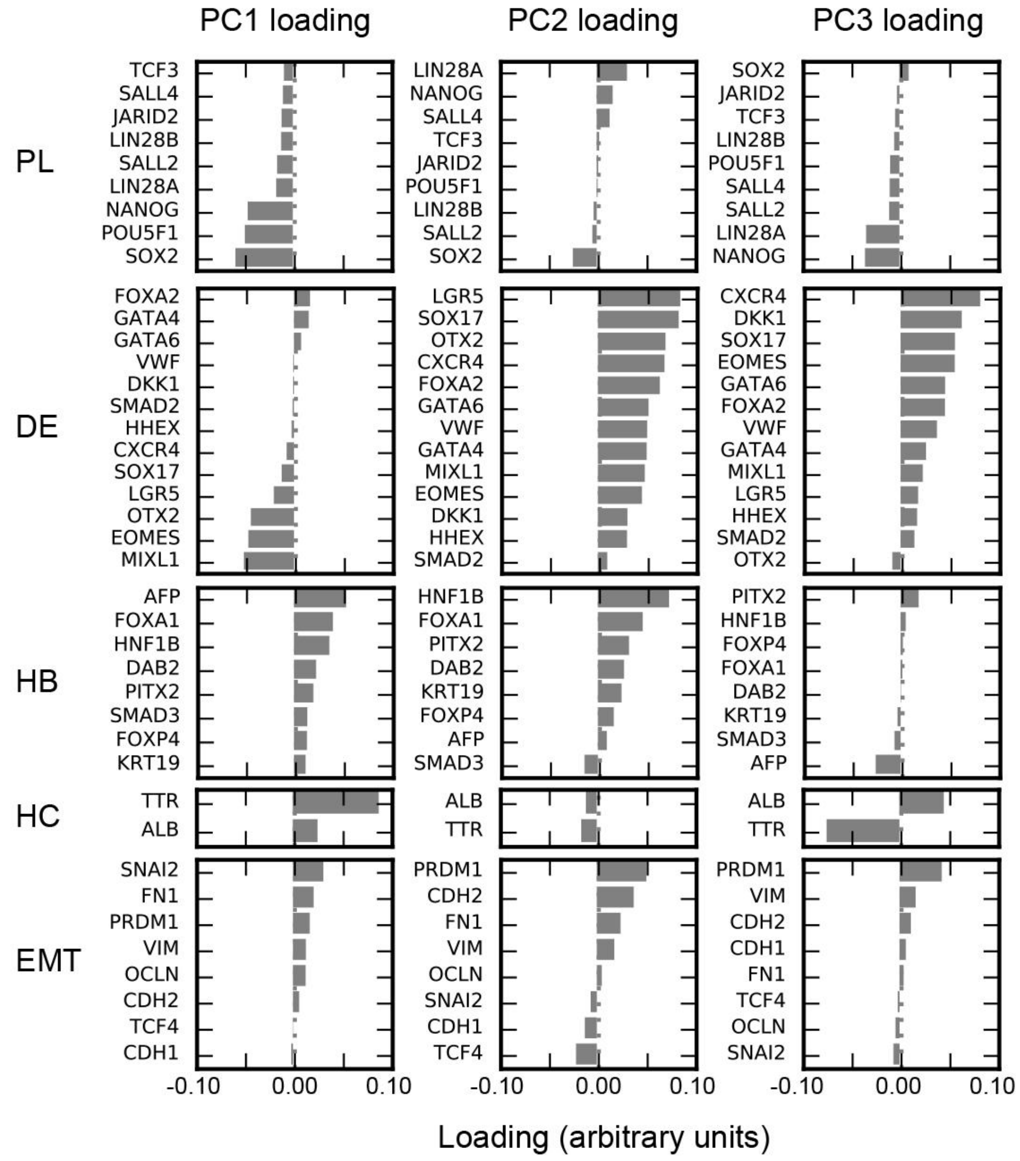

Supplementary Figure 2, Gene loading for PCs of Figure 1b

Supplementary Figure 3

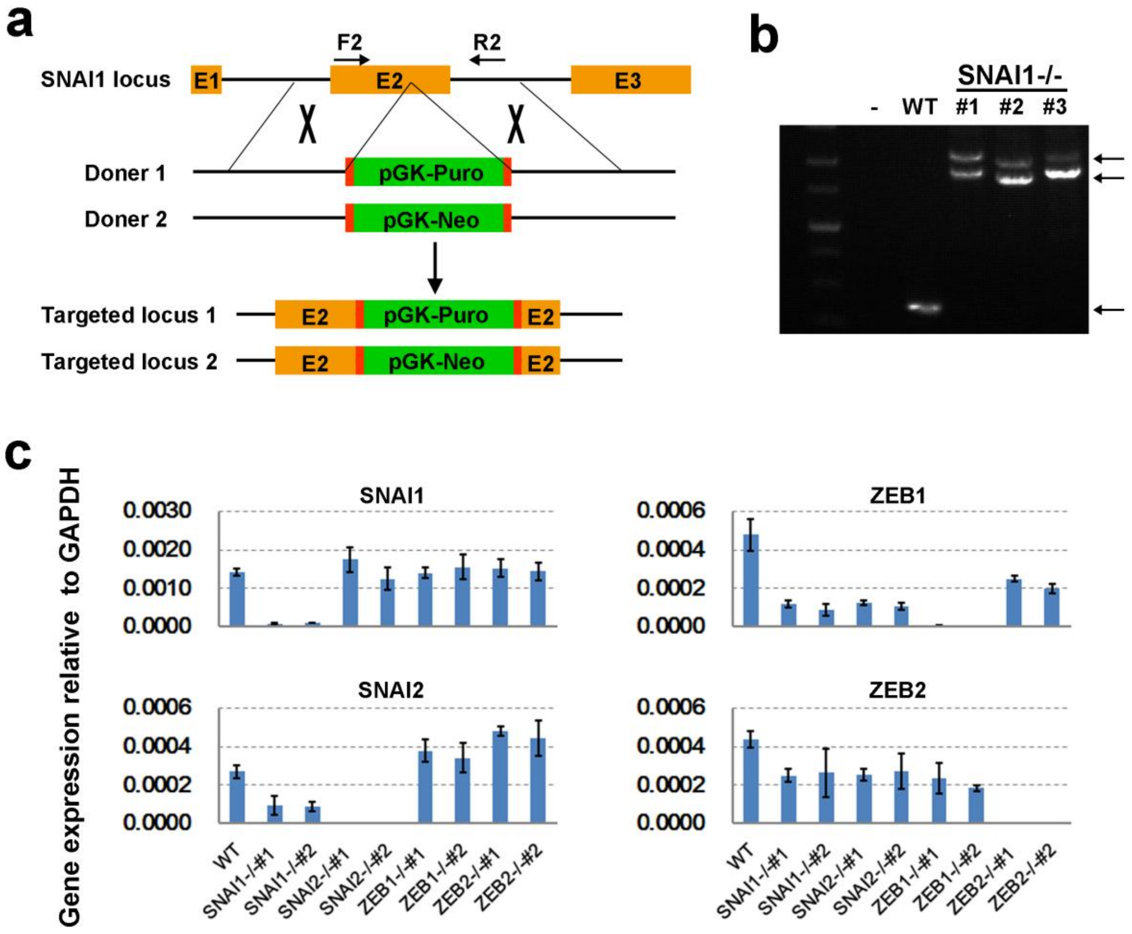

Supplementary Figure 3. Strategy and validation of gene targeting in H1 cells

(a) The whole scheme of SNAI1 knockout experiment design. (E2: exon 2)

(b) PCR validation on genomic DNA with the primer F2 and R2. The 350 bp product for wild type allele and 1.7/2.0 Kb fragments for pGK-Puro/Neo targeted integration suggested a biallele SNAI1 knockout.

(c) qRT-PCR analysis of SNAI1/2 and ZEB1/2 expression at D3 further confirmed the gene targeting effect. SNAI1/2 and ZEB1/2 genes were silenced respectively in their own knock-out clones but not the other ones. Data represent mean ±s.d. from three independent repeats.

Supplementary Table 1, list of antibodies used in this study

Name of Antibody	Company（Cat. No.）	Dilution Factor
Rabbit anti E-cadherin antibody	ABclonal Biotechnology (A0965)	1:100
Mouse anti N-cadherin antibody, clone 32	BD Bioscience (610920)	1:100
Rabbit anti N-Cadherin (D4R1H) antibody	Cell Signaling Technology(13116)	1:200
Goat anti SOX17 antibody	R&D systems (AF1924)	1:400
Mouse anti Oct-3/4 antibody, clone C-10	Santa Cruz Biotechnology (sc-5279)	1:50
Rabbit anti Oct-4A antibody, clone C30A3	Cell Signaling Technology(2840)	1:200
Rabbit anti Nanog antibody,clone D73G4	Cell Signaling Technology(4903)	1:200
Goat anti ALB antibody	GeneTex (GTX19180)	1:100
Rabbit anti HNF4 alpha antibody, clone EPR3648	GeneTex (GTX62347)	1:100
Rabbit anti Cytochrome P450 antibody	Abcam (ab62204)	1:200
Goat anti AAT antibody	Santa Cruz Biotechnology (sc-14586)	1:100
Mouse anti Snail antibody, clone L70G2	Cell Signaling Technology（3895)	1:100
Rabbit anti Vimentin (R28) Antibody	Cell Signaling Technology(3932)	1:50
Mouse anti AFP antibody	GeneTex (GTX84948)	1:200
Mouse anti PAX6 antibody	DSHB(AB_528427)	1:500
Rabbit anti PAX6 antibody	Sigma(HPA030775)	1:100
Alexa Fluor® 488 Donkey Anti-Mouse IgG (H+L) Antibody	Invitrogen (A21202)	1:400
Alexa Fluor® 488 Donkey Anti-Rabbit IgG (H+L) Antibody	Invitrogen (A21206)	1:400
Alexa Fluor® 488 Donkey Anti-Goat IgG Antibody	Invitrogen (A11055)	1:400
Alexa Fluor® 568 Donkey Anti-Mouse IgG Antibody	Invitrogen (A10037)	1:400
Alexa Fluor® 568 Donkey Anti-Rabbit IgG Antibody	Invitrogen (A10042)	1:400

Supplementary Table 2, list of primers for qRT-PCR

Gene	Forward primer	Reverse primer
SOX2	CCCAGCAGACTTCACATGT	CCTCCCATTTCCCTCGTTTT
EOMES	CCGCCACCAAACTGAGATGA	ACATTTTGTTGCCCTGCATGT
SOX17	CGCACGGAATTTGAACAGTA	GGATCAGGGACCTGTCACAC
FOXA2	ACTACCCCGGCTACGGTTC	AGGCCCGTTTTGTTCGTGA
LGR5	CTCCCAGGTCTGGTGTGTTG	GAGGTCTAGGTAGGAGGTGAAG
GSC	AACGCGGAGAAGTGGAACAAG	CTGTCCGAGTCCAAATCGC
PAX6	ATGTGTGAGTAAAATTCTGGGCA	GCTTACAACTTCTGGAGTCGCTA
CDH1	TGCCCAGAAAATGAAAAAGG	GTGTATGTGGCAATGCGTTC
CDH2	TGGGAATCCGACGAATGG	TGCAGATCGGACCGGATACT
KLF8	CCCAAGTGGAACCAGTTGACC	GACGTGGACACCACAAGGG
SNAI1	ACTGCAACAAGGAATACCTCAG	GCACTGGTACTTCTTGACATCTG
SNAI2	CATGCCTGTCATACCACAAC	GGTGTCAGATGGAGGAGGG
ZEB1	AGCAGTGAAAGAGAAGGGAATGC	GGTCCTCTTCAGGTGCCTCAG
VIM	GACGCCATCAACACCGAGTT	CTTTGTCGTTGGTTAGCTGGT
TGFB1	CTAATGGTGGAAACCCACAACG	TATCGCCAGGAATTGTTGCTG
OTX2	GGAAGCACTGTTTGCCAAGACC	CTGTTGTTGGCGGCACTTAGCT
POU5F1	CCTCACTTCACTGCACTGTA	CAGGTTTTCTTTCCCTAGCT
NANOG	TGAACCTCAGCTACAAACAG	TGGTGGTAGGAAGAGTAAAG
AFP	ATTGGCAAAGCGAAGCTG	GCTGTGGCTGCCATTTTT
ALB	GGTGTTGATTGCCTTTGCTC	CCCTTCATCCCGAAGTTCAT
SNAI1-KO	AGATGCACATCCGAAGCCACAC	GACATCTGAGTGGGTCTGGAGGTG
SNAI2-KO	GGATACTCCTCATCTTTGGGGCG	CCAGCCCAGAAAAAGTTGAATAGGTC
ZEB1-KO	TGCTCCCTGTGCAGTTACACCTTT	CAGTTTGGGCATTCATATGGCTTC
ZEB2-KO	CTGCCACCTGGAACTCCAGATG	TCTCTTCATTCTTCTCGTGGCGG
GAPDH	AGGGCTGCTTTTAACTCTGGT	CCCCACTTGATTTTGGAGGGA

Supplementary Table 3, list of Taqman primers used for single cell qPCR

Human gene name	Human Taqman	Note
CXCR4	Hs00607978_s1	Early endoderm
EOMES	Hs00172872_m1	Early endoderm
HHEX	Hs00242160_m1	Early endoderm
KIT	Hs00174029_m1	Early endoderm
MIXL1	Hs00430824_g1	Early endoderm
SOX17	Hs00751752_s1	Early endoderm
OTX2	Hs00222238_m1	Early endoderm/ectoderm
EN1	Hs00154977_m1	Ectoderm marker
OLIG1	Hs00744293_s1	Ectoderm marker
PAX6	Hs00240871_m1	Ectoderm marker
SOX1	Hs01057642_s1	Ectoderm marker
TP63	Hs00978344_m1	Ectoderm marker
CDH2	Hs00983056_m1	Mesenchymal marker
KLF8	Hs00604465_m1	Epithelial marker
AFP	Hs00173490_m1	Hepatocyte
ALB	Hs00910225_m1	Hepatocyte
IGDCC4	Hs00326335_m1	Hepatocyte
TTR	Hs00174914_m1	Hepatocyte
HNF4A	Hs00230853_m1	Hepatocyte precursor
KRT19	Hs00761767_s1	Hepatocyte precursor
FOXA2	Hs00232764_m1	Endoderm
GATA4	Hs00171403_m1	Endoderm
GATA6	Hs00232018_m1	Endoderm
SMAD3	Hs00969210_m1	Endoderm
ACTB	Hs01060665_g1	Loading Control
GAPDH	Hs02758991_g1	Loading Control
CDH1	Hs01023894_m1	Epithelial marker
LGR5	Hs00969422_m1	Endoderm
PRDM1	Hs00153357_m1	Mesenchymal marker
SNAI1	Hs00195591_m1	Mesenchymal marker

SNAI2	Hs00950344_m1	Mesenchymal marker
TCF4	Hs00162613_m1	Mesenchymal marker
ZEB1	Hs00232783_m1	Mesenchymal marker
FN1	Hs00365052_m1	Mesenchymal marker
OCLN	Hs00170162_m1	Epithelial marker
VIM	Hs00185584_m1	Mesenchymal marker
GSC	Hs00906630_g1	Mesenchymal marker
GATA2	Hs00231119_m1	Mesoderm marker
HAND1	Hs02330376_s1	Mesoderm marker
HAND2	Hs00232769_m1	Mesoderm marker
KDR	Hs00911700_m1	Mesoderm marker
NKX2-5	Hs00231763_m1	Mesoderm marker
T	Hs00610080_m1	Mesoderm marker
NANOG	Hs04260366_g1	Pluripotency
POU5F1	Hs04260367_gH	Pluripotency
SOX2	Hs01053049_s1	Pluripotency/Neurectoderm
FGF17	Hs00915256_m1	Endoderm
CER1	Hs00193796_m1	Endoderm

Supplementary Table 4, list of sgRNAs and primers for gene targeting

Gene	sgRNA sequence	Position	Primers for insert validation		Primers for donor DNA construction	
SNAI1	GGCACGTACCAGTGTGGGTC	exon 2	SNAI1-F2	AGGATCTCCAGGCTCGAAAGGC	left arm-F	GTTCTTCTGCGCTACTGCTGCG
					left arm-R	ATGGCCTTGTAGCAGCCAGGG
			SNAI1-R2	ACGTTTCCAGAGAGCCAGGCC	right arm-F	CCACACTGGTACGTGCCCCTC
					right arm-R	TGCCAGTCCTCCCAGACACCTC
SNAI2	GGGTCCGAATATGCATCTTC	exon 2	SNAI2-F2	AGGAGCATACAGCCCCATCACTG	left arm-F	AAACGGGCTCAGTTCGTAAAGGAG
					left arm-R	AGTCTTTCCTCTTCATCACTAATGGGG
			SNAI2-R2	ACATGCTGTTTGCAGTCCCTGG	right arm-F	CGGCAAGGCGTTTTCCAGAC
					right arm-R	TCCATGCTCTTGCAGCTCTCTCTC
ZEB1	AGAGACATGTGACGCAGTCT	exon 6	ZEB1-F2	ACAGGTTATTCAGTATTTGTGGATGCG	left arm-F	GACGGAGCTGGCTTGCAGGAG
					left arm-R	CATTTGAATTTACGATTACACCCAGACTG
			ZEB1-R2	CCCTGCTAATTGTGCAAAGGAGG	right arm-F	AGCTTTCAAATACAAACATCACCTAAAAGAG
					right arm-R	AAGAAACAGAATATTGTGGCAGGCTG
ZEB2	TTGTAGCCCCGGTCGCAGTA	exon 5	ZEB2-F2	ACGCCCCTTCACACTGTGAGTTC	left arm-F	TCCTGGCTGTCTGTGACATTCAGAG
					left arm-R	TTGGGCAAAAGCATCTGGAGTTC
			ZEB2-R2	AAACCATCCCCCCACCTGATC	right arm-F	AAGAATGAAGAGAACTTTTCCTGCCC
					right arm-R	TGAAAAGCATTCAGGTGACAAGCC
